# Evidence of Inverse Hall-Petch Behavior and Low Friction and Wear in High Entropy Alloys

**DOI:** 10.1038/s41598-020-66701-7

**Published:** 2020-06-23

**Authors:** Morgan R. Jones, Brendan L. Nation, John A. Wellington-Johnson, John F. Curry, Andrew B. Kustas, Ping Lu, Michael Chandross, Nicolas Argibay

**Affiliations:** Material, Physical, and Chemical Sciences Center, Sandia National Laboratories, Albuquerque, NM 87123 USA

**Keywords:** Structural materials, Structural properties

## Abstract

We present evidence of inverse Hall-Petch behavior for a single-phase high entropy alloy (CoCrFeMnNi) in ultra-high vacuum and show that it is associated with low friction coefficients (~0.3). Grain size measurements by STEM validate a recently proposed dynamic amorphization model that accurately predicts grain size-dependent shear strength in the inverse Hall-Petch regime. Wear rates in the initially soft (coarse grained) material were shown to be remarkably low (~10^–6^ mm^3^/N-m), the lowest for any HEA tested in an inert environment where oxidation and the formation of mixed metal-oxide films is mitigated. The combined high wear resistance and low friction are linked to the formation of an ultra-nanocrystalline near-surface layer. The dynamic amorphization model was also used to predict an average high angle grain boundary energy (0.87 J/m^2^). This value was used to explain cavitation-induced nanoporosity found in the highly deformed surface layer, a phenomenon that has been linked to superplasticity.

## Introduction

Since their discovery in 2004^1,2^, high entropy alloys (HEAs) have been extensively investigated and shown to exhibit remarkable thermomechanical properties^3,4^. Studies of grain-size dependent mechanical behavior are limited^5,6^, however, especially in the regime of ultra-nanocrystalline grain size where softening (i.e. inverse Hall-Petch behavior) occurs. Recent work has also shown that these alloys are ideally suited for processing using laser-based additive manufacturing techniques^7–9^, as they exhibit high phase stability and derive much of their strength from thermal history-insensitive mechanisms like solution strengthening^10^. While recent publications have presented experimental evidence of low friction or high wear resistance (but typically not both) with HEAs^11–25^, they were performed in environments where oxidation and the formation of mixed metal-oxide surface films was prevalent. These phenomena can greatly impact strength and deformation/wear resistance. We show that in an ultra-high vacuum (UHV) environment, i.e., in the absence of chemically reactive species, an additively manufactured initially coarse grained (soft), single-phase HEA exhibited a tendency toward low friction coefficients (i.e., low shear strength) and some of the lowest wear rates currently reported for these materials. The low friction and wear are attributed to a propensity for extreme grain refinement under sliding contact. Specifically, we present evidence of that this high strain rate shear leads to dynamic amorphization and inverse Hall-Petch behavior in the surface layer. In the present context of sliding contacts, dynamic amorphization refers to the continuous shear-induced generation of a structurally amorphous layer that accommodates deformation^26^. This is in competition with stress- and temperature-driven grain growth and recrystallization, all of which reestablish crystallinity and order in the shear layer. The shear layer must be amorphized again in subsequent contact passes, thus we refer to this process as dynamic amorphization.

## Results and Discussion

**Figure 1:**
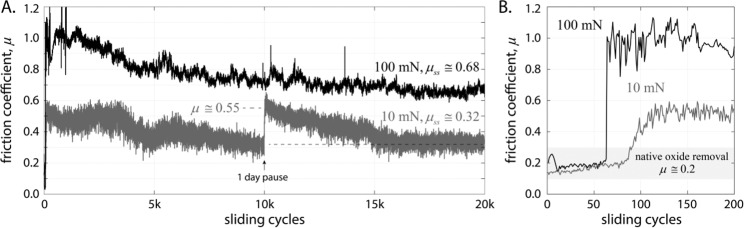
(**A**) Average friction coefficient per sliding cycle for additively manufactured single-phase CoCrFeMnNi sliding against ruby spheres in UHV (~10^–9^ torr) at 1 mm/s; (**B**) magnified view of the first 200 cycles of sliding showing the early cycle-dependent transition in friction behavior.

Sliding contact experiments were performed on an equiatomic CoCrFeMnNi high entropy alloy bulk specimen, with an average initial grain size of approximately 42 µm, that was fabricated by laser 3D printing of gas atomized powder^9^. These experiments were performed in UHV (~10^–9^ torr) to mitigate the impact of oxidation and adsorbates. By comparison to previous literature, we show that environment can have a significant and detrimental impact on the friction and wear properties of this alloy. Representative sliding cycle-average friction coefficients are shown in Fig. 1A for two contact loads, 10 mN and 100 mN, for highly smooth, ruby spheres (Al_2_O_3_, with initial RMS surface roughness ~ 10 nm) sliding on the samples. In the low friction case, the experiments were paused mid-way for 24 hours to help elucidate the role of microstructural evolution on interface strength, and thus friction coefficient; this is discussed in more detail, below.

Average steady-state friction coefficients of ${\mu }_{ss}\cong 0.32$ and ${\mu }_{ss}\cong 0.68$ were measured at 10 and 100 mN, respectively. In the absence of oxides and adsorbates, the friction coefficient of metals is a measure of the surface shear strength of the metal, and this has been directly correlated to grain size^27–29^. For sliding metal-on-metal or ceramic-on-metal contacts (noting that metals are softer than ceramics like ruby) the steady-state friction coefficient can be estimated as the ratio of surface shear strength and load-bearing (bulk) hardness $\mu \cong \frac{{\tau }_{surf}}{{H}_{bulk}}.$^30^. It is widely reported that deformation due to sliding contact of metals is confined to a thin surface layer^28,29,31–37^, typically about 100 nm, whereas the volume that participates in supporting the normal force is much larger^38^ even at relatively mild contact stresses like those used here. The bulk hardness or flow stress can be converted into a shear strength using the approximation $\tau \cong \frac{H}{3\sqrt{3}}$, allowing a comparison between shear strength and hardness as a function of grain size^26^. Figure 1B shows a magnified view of the first 200 cycles for both experiments, showing the transition from sliding on – and removal of – the native oxide to the bulk metal. Sliding on initially coarse-grained metal leads to rapid, severe plastic deformation^39^, and as we elaborate below, the load dependent transition to different deformation regimes and shear strengths leads to a divergence in friction behavior. However, the initial 50 cycles exhibit the same friction coefficient, indicating that prior to the divergence in shear strength, the friction coefficient was independent of applied load, obviating plowing due to excessive load as a reason for the divergence^40–43^.

**Figure 2:**
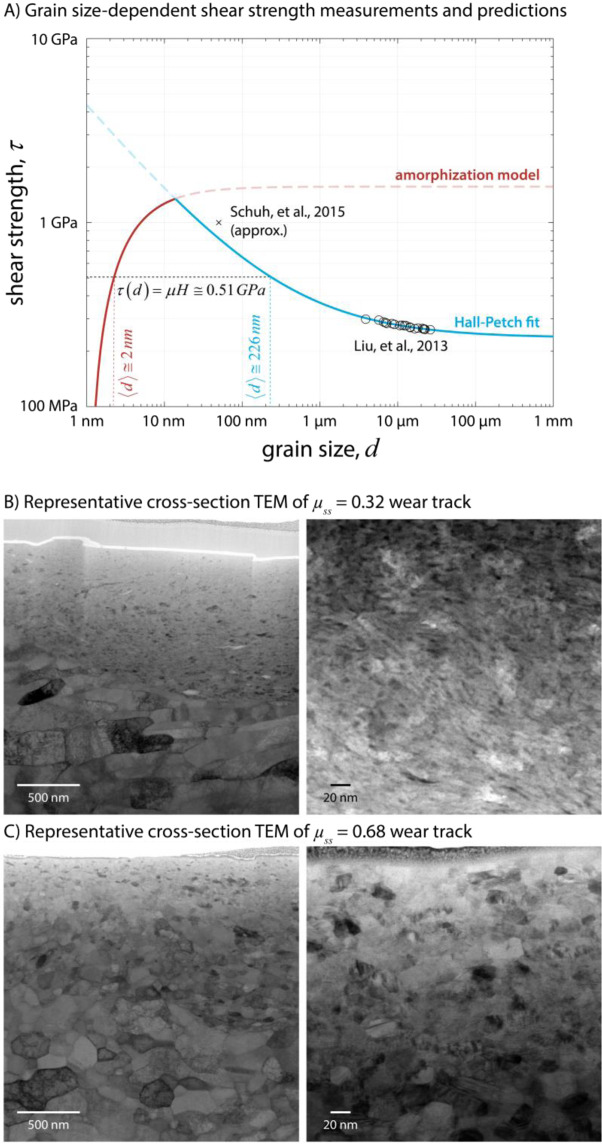
Plot of grain size-dependent shear strength data for equiatomic CoCrFeMnNi from Liu, *et al*.(open circles)^6^, a fit of this data to the Hall-Petch equation (blue line), an estimate of strength in the nanocrystalline regime from Schuh, *et al*.^5^ (x), and a prediction of strength due to dynamic amorphization (red line);^26^ (B and C) show representative cross-sectional STEM images along wear track centerlines comparing near-surface grain sizes for (**B**) the low friction case, with evidence of grains smaller than 10 nm, and (**C**) the high friction case with measured average grain size approximately 17 + /- 6.5 nm. The STEM images are all brightfield except for the right image in B, which is darkfield, to better highlight nanoscale voids (dark spots).

A plot of grain size-dependent shear strength for CoCrFeMnNi is shown in Fig. 2, including data from a systematic investigation of Hall-Petch behavior from Liu, *et al*.^6^ and an estimate in the nanocrystalline regime (grain size ca. 80 nm) from a torsional severe plastic deformation (SPD) investigation by Schuh, *et al*.^5^. The Hall-Petch equation, ${\sigma }_{y}={\sigma }_{0}+k\cdot {d}^{-1/2}$, is fit to the Luo data only, and agrees reasonably well with the nano-grained estimate from Schuh, *et al*.^5^.

Recent work has shown that low friction of bare metals, in the range $\mu \cong 0.2-0.5$, can be explained as the dynamic softening (i.e. inverse Hall-Petch behavior) of surfaces due to highly surface-localized, shear-induced severe plastic deformation^28,29,31–34^. This is well described by a recently proposed predictive model^26^ that explains the low shear strength and friction coefficients of bare metals and alloys. This model of grain size-dependent shear strength requires no fitting parameters and is based exclusively on materials properties and physically meaningful input parameters like temperature and shear rate. Here, we show that this model can also accurately explain the friction behavior of a high entropy alloy, and by doing so presents evidence of inverse Hall-Petch behavior, or softening, due to extreme grain refinement for high entropy alloys. When the surface grain size is dynamically refined below a critical grain size, ${d}_{crit}\cong 10-50\,nm$ for most metals, the role of dislocations and intragranular deformation is suppressed and grain boundary sliding mechanisms become energetically favorable^26,29,44–46^.

The bulk hardness was determined to be 1.6 GPa by Vickers indentation. This hardness was used to estimate the shear strengths associated with the friction coefficients shown in Fig. 1 through the relation $\tau (d)\cong \mu H$. Comparison to Fig. 2A then gives a prediction of the average dynamic grain size via the amorphization model. As is shown in Fig. 2A, two possible grain sizes can be associated with a steady-state friction coefficient ${\mu }_{ss}=0.32$ ($\tau \cong 0.51\,GPa$), $d\cong 2\,nm$ or $d\cong 226\,nm$. Scanning transmission electron microscope (STEM) images of the lower friction wear track (Fig. 2B) show that while the size is bimodal (discussed in more detail below), the grains are clearly smaller than 20 nm, eliminating the larger grain size as a possibility. As naturally occurring grain growth and recrystallization (mechanisms that compete with refinement) during processing and post-test analysis of such highly refined regions is expected^5,47–49^, only the 2–3 nm average grain size is a reasonable conclusion. The STEM images are not to be taken as representative of the grain size during sliding, but rather only as clear evidence that the *in situ* grain size could not have been *greater* than those observed in the post-test analysis, ruling out the classic Hall-Petch regime in the low friction case. This information is crucial to the determination of the shear strength mechanism, allowing the subsequent prediction of grain size dependent shear strength (and thus an estimation of friction coefficient) in the inverse Hall-Petch regime. The higher resolution image in Fig. 2B was taken near the surface, showing approximately 10 nm size grains interspersed with amorphous metal, and most importantly, ruling out the 200+ nm grain size that corresponds to the Hall-Petch regime.

Estimation of grain size for the low friction case from STEM micrographs (Fig. 2B) was particularly difficult since the near-surface grain size appears to consist of a bimodal distribution of near-amorphous and nanocrystalline grains. The average size for measurable grains was *d* = 13 + /− 5 nm, though this is an upper bound as it ignores large regions where grains were too small to measure. The few dispersed grains that were measurable were equiaxed and dispersed in a near-amorphous matrix, indicative of recrystallization and grain growth having occurred after sliding. A one day pause in sliding (with the probe retracted and out of contact) at the 10k cycle mark for the low friction case, shown in Fig. 1, was used to highlight this phenomenon. Upon resuming sliding, the friction coefficient increased to $\mu \cong 0.55$, and gradually, over thousands of additional sliding cycles, returned to the same steady-state value of ${\mu }_{ss}\cong 0.32$. With a hardness of 1.6 GPa, $\mu \cong 0.55$ corresponds to a shear strength $\tau \cong 0.88\,GPa$, implying the grains grew to an average size of about 5 nm during the pause, in good agreement with the post-sliding TEM analysis. Approximately 11 days elapsed between the completion of an experiment and TEM analysis. Grain growth kinetics are typically non-linear and grain size-dependent^49^, leaving ample time for relaxation and grain growth as evident in TEM images. The dark contrast regions on the order of a few nm in Fig. 2B are voids attributed to cavitation. As voids can be more thermodynamically stable than similarly sized grains^50–52^, they are likely an indication of finer grain size present during sliding. *Ex situ* techniques like nanoindentation and electron microscopy are not an ideal way to determine the grain size of ultra-nanocrystalline surface shear layers during sliding contact for most metals, as these layers are extremely thin and naturally predisposed to rapid grain growth and recrystallization.

For the higher friction case, with steady-state friction coefficient ${\mu }_{ss}\cong 0.68$, the estimated shear strength, $\tau (d)=(0.68)(1.6\,GPa)=1.1\,GPa$, corresponding to an average grain size in the range 10–20 nm (i.e. at the peak of the Hall-Petch curve, where shear is accommodated both through dislocations and grain boundary sliding). Grains of this size are more easily measured via *ex situ* TEM. The representative TEM images shown in Fig. 2C confirm this estimate, with an average grain size (determined by the line intercept method) of *d* = 17.3 + /- 6.5 nm, in excellent agreement with the prediction from Fig. 2. Note that for this sample, the average initial grain size was approximately 42 µm^9^.

**Figure 3:**
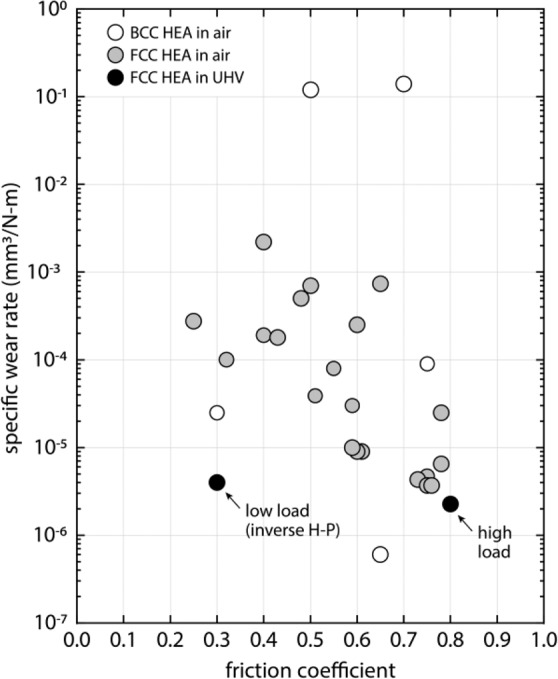
Literature survey of steady-state friction coefficients and wear rates for HEAs tested in ambient conditions^11–25^, and the current results for single-phase CoCrFeMnNi in UHV.

The specific wear rate, defined as the ratio of volume lost to the product of applied normal force and sliding distance, $K=\frac{V}{{F}_{n}\cdot d}$, was calculated for the two experiments shown in Fig. 1. The volume lost was determined using a high-resolution topographical interferometric scan of the entire length of each wear track. In Fig. 3 we show the present results compared to a summary of published steady-state friction coefficients and wear rates for a wide range of high entropy alloys. In the case of multi-phase alloys, these are differentiated as FCC- or BCC-based, corresponding to the primary phase. In all but the present case, the data correspond to experiments performed in environments with sufficient oxygen concentration to drive the formation of mixed metal-oxide films^11–25^. A conclusion of this work is that, when sheared in UHV, the soft, single-phase (FCC), initially coarse grained (42 µm) CoCrFeMnNi specimens used in this investigation showed nearly the lowest wear rates. This is surprising given that shear deformation of HEAs, even high hardness (BCC) and multi-phase (FCC-BCC) alloys, generally shows rapid formation of mixed metal-oxide surface films that tend to reduce wear rate^14,53^ and increase hardness^54^. Remarkably, the higher friction and contact load case showed comparable or even slightly lower wear rate compared to the low friction case. These wear rates are likely a further indication that sliding shear in these alloys occurred primarily through the formation of an amorphous interface and subsequent grain boundary sliding^27^. Generally, the measured friction coefficients and wear rates are comparable to other nanocrystalline alloys, in the absence of oxide films and other surface modifiers, with values as low as $\mu \cong 0.2$ and $K\cong {10}^{-6}-{10}^{-9}\frac{m{m}^{3}}{N-m}.$^27,29,55^.

In addition to describing the shear strength, the dynamic amorphization model can also be used to make a prediction of the average high angle grain boundary (HAGB) energy for this alloy^26^, using the expression,

1 $${\gamma }_{HAGB,calc}=\left(L\frac{{\rho }_{L}}{M}\right)\left(1-\frac{T}{{T}_{m}}\right)(2b)$$

This requires the use of materials parameters from the literature for this equiatomic high entropy alloy composition, with heat of fusion^56^, L = 16.2 kJ/mol, melting temperature^57^, *T*_*m*_ = 1,680 K, liquid density at the melting temperature, ${\rho }_{L}\cong 7,200\,\frac{kg}{{m}^{3}}$, molar mass, $M=\frac{{\rho }_{RT}{A}_{v}{a}^{3}}{4}\cong \frac{\left(8000\frac{kg}{{m}^{3}}\right)\left(6.022x{10}^{23}\frac{atoms}{mol}\right){(0.36nm)}^{3}}{4}=0.056\frac{kg}{mol}$, room temperature density^9,58^, ${\rho }_{RT}\cong 8,000\frac{kg}{{m}^{3}}$, lattice parameter^59^, $a=0.36\,nm$, and atomic diameter, $b=\frac{a}{\sqrt{2}}.$ The liquid density was estimated as, ${\rho }_{L}\cong 0.9{\rho }_{RT}$, a relationship that is generally accurate for metals.

This calculation leads to a prediction for the average high angle grain boundary energy (at 300 K), ${\gamma }_{HAGB,calc}\cong 0.87\,\frac{J}{{m}^{2}}$. Although recent experimental measurements of HAGB energy with this same alloy (CoCrFeMnNi) exist^60^, a comparison of this prediction is difficult. Measurements of grain boundary energy are sensitive to thermally-driven grain boundary segregation (and possibly phase separation) of impurities^61^. This value is relatively high for metals^26,62^, and may imply a propensity for localized cohesive failure or fracture at the boundaries^63^. Grain boundary decohesion has been recently shown to occur readily for this alloy due to segregation and nanoclustering of Cr, Ni and Mn at elevated temperature^64^, and these failures typically originate along HAGBs and at triple junctions^65^. In the present work there is no significant evidence of grain boundary segregation, and this is reasonable as a negligible increase in temperature is expected for these sliding conditions^66^. This suggests that this alloy may also be prone to grain boundary decohesion without significant segregation and phase separation, i.e., when it is a solid solution. This phenomenon is sometimes discussed as cavitation in the superplasticity literature, where grain boundary sliding mechanisms similarly dominate^67^, and suggests the possibility that low friction and high wear resistance may be evidence of low temperature and high strain rate superplasticity. In other words, low friction is essentially high ductility without failure and the concomitant rapid generation of wear particles.

This work studied an equiatomic CoCrFeMnNi high entropy alloy that was manufactured using a laser-based consolidation additive manufacturing process. We showed that low friction and high wear resistance are achievable with bare high entropy alloys in inert environments. These characteristics are linked to the shear-induced formation of a highly refined, near-amorphous surface film that exhibits inverse Hall-Petch behavior. The detrimental role of environments, specifically those that result in oxidation, was mitigated by tribological testing in a UHV environment. A newly proposed amorphization model was used to accurately predict the grain size-dependent shear strength, and thus friction coefficient.

## Methods

### Sample preparation

Pre-alloyed powder of the equiatomic CoCrFeMnNi alloy was prepared using high-pressure gas atomization (HPGA) of 18.4 kg of pre-alloyed (Fe-Mn, Ni-Cr) and elemental (Co) charge materials in a bottom pour zirconia crucible arrangement. An induction furnace was used to heat the charge materials to ~1900 °C. The chamber atmosphere was first evacuated to 26 Pa, then backfilled to 6.89 kPa Ar (99.999% purity) over-pressure during atomization. Atomization was performed using 1.09 MPa Ar (99.999% purity) gas. Powders were screen classified according to ASTM B214-16. Specimens were constructed in this study using the 45–75 µm powder size.

3D printing of the CoCrFeMnNi alloy powder was performed using an open architecture Laser Engineered Net Shaping (LENS) system that utilized a 2 kW fiber laser emitting at 1064 nm wavelength. The laser was mounted to the spindle of a Tormach PCNC 770 milling machine platform placed inside a glove box^68^. During processing, an inert atmosphere was maintained, with <50ppm O_2_ and <10ppm H_2_O, by continuously flowing Ar gas. A range of laser powers (350–400 W) and build velocities (400–600 mm/min) were used to construct the specimens. The build layer thicknesses was 250–300 µm with a constant hatch spacing of 800 µm.

Samples were machined into 25 mm ×12 mm ×5 mm coupons. Specimens were ground using SiC paper and then polished with a 3 µm MOL polishing cloth, resulting in an average surface roughness of R_a_ ~ 250 nm.

### Tribological testing

A deadweight load rotary tribometer was used to perform friction measurements. The tribometer, like that described previously^69,70^, was operated at room temperature inside of an ultra-high vacuum (UHV) chamber with chamber pressures ~10^–9^ torr during testing. Two loading conditions were employed to study the stress-dependent friction behavior of the CrCoFeMnNi alloy: 10 mN and 100 mN. For each experiment, a 3.2 mm diameter sapphire sphere was run at an angular velocity of ~ 15.5 rpm with a 45-degree stroke length. Tests ran for at least 20k bidirectional sliding cycles, with individual tests being stopped and restarted every 10k cycles to study hysteresis and grain growth effects. During the periods of stop-time, the UHV tribometer was kept under vacuum, to ensure the sample was not exposed to air. Friction force data was acquired at 10 kHz, and the average friction coefficient (µ) was calculated as the ratio of the measured friction force and applied deadweight (normal) load.

### Microscopy

A Bruker Contour GT-I optical interferometer was used at a 10x objective and 1x magnification (lateral resolution of ~ 1 µm and height resolution <1 nm) to topographically scan the entirety of the wear track. The specific wear rates (*K*) were calculated based on average wear track cross-sections using the expression, $K=\frac{A}{{F}_{n}\cdot N}\cdot {10}^{3}$, where A is the average worn cross-sectional area, *F*_*n*_ is normal force, and *N* is number of cycles^71^. The associated uncertainty in reported specific wear rate is discussed elsewhere^72^.

TEM samples from the wear tracks were made by focused ion beam (FIB). An FEI Titan G2 80–200 STEM with a Cs probe corrector and ChemiSTEM technology (X-FEG and SuperX EDS with four windowless silicon drift detectors) operated at 200 kV was used in this study. The microstructure was studied by STEM using bright-field detector of the collection range of 0–30 mrad as well as high-angle annular dark-field (HAADF) detector of the collection range of 70–160 mrad. Combination of BF and HAADF imaging allows the grain structure to be better visualized.
